# Study of Lactic Acid Bacteria Biodiversity in Fermented *Cobrançosa* Table Olives to Determine Their Probiotic Potential

**DOI:** 10.3390/foods11193050

**Published:** 2022-10-01

**Authors:** Joana Coimbra-Gomes, Patrícia J. M. Reis, Tânia G. Tavares, Francisco Xavier Malcata, Angela C. Macedo

**Affiliations:** 1 LEPABE—Laboratory for Process Engineering, Environment, Biotechnology and Energy, Faculty of Engineering, University of Porto, Rua Doutor Roberto Frias, 4200-465 Porto, Portugal; 2 ALiCE—Associate Laboratory in Chemical Engineering, Faculty of Engineering, University of Porto, Rua Doutor Roberto Frias, 4200-465 Porto, Portugal; 3 UNICES—Research Unit in Management Sciences and Sustainability, University of Maia, Avenida Carlos de Oliveira Campos, 4475-690 Maia, Portugal

**Keywords:** fermentation, *Lactiplanctibacillus* spp., health, food safety, functional food, gastrointestinal tract, gut survival, probiotic, starter culture

## Abstract

Current market trends point at increasing demand for functional foods, namely those carrying probiotics. In the case of table olives, presence of probiotics would convey a competitive advantage to Mediterranean-based diets, already established for their cultural heritage and gastronomic character. This work assessed the safety and resistance to gastrointestinal digestion of 19 native LAB strains from *Cobrançosa* table olives. Strains were identified via molecular sequencing (4 fingerprints/10 strains for *Lactiplantibacillus* *pentosus*, and 2 fingerprints/9 strains for *L. paraplantarum*), and exposed to simulated gastrointestinal fluids, as per the INFOGEST in vitro protocol with modifications. None of those strains proved dangerous for human consumption. Survivability to the gastrointestinal resistance test ranged from 29% to 70%, with strain-dependent variability. *L. paraplantarum* i18, i27, and i102, and *L. pentosus* i10 and i11 exhibited statistically lower survival rates (29–35%) than probiotic the Greek table olive reference strain *L*. *pentosus* B281 (53%). Among the other strains, *L. paraplantarum* i101 and *L. pentosus* i53 and i106 showed the highest survival rates but were not significantly different from the strain of *Lacticaseibacillus casei* isolated from commercial probiotic yoghurt (65–70%). In vitro results proved that strains retrieved from fermenting cultivar *Cobrançosa* possess the potential to be claimed as probiotics—thus deserving further attention toward the development of a specific starter culture.

## 1. Introduction

In order to remain competitive in the global market, agri-food industries have been struggling for innovation in both process and product—while trying to address the issues of environmental sustainability and food safety [1]. Therefore, stakeholders are evolving from conventional approaches to food production to the addition of vectors for the enhancement of bioavailability of ingredients responsible for health benefits—further to satisfy basic nutritional needs. Such functional products include probiotic- and prebiotic-containing foods, as well as dietary supplements, vitamins, antioxidants, and fiber as nutraceutical ingredients [2,3].

A major window of opportunity has thus arisen in the field of novel probiotic strains—isolated from less-conventional natural sources—able to confer advanced health promoting properties to the host upon regular and balanced administration via traditional foods; this includes adventitious strains isolated from plant matrices, in alternative to classical, animal-based probiotic strains in dairy foods [4,5].

Fermented table olives hold a nuclear role in the Mediterranean diet, and accordingly rank among the most popular and healthy fruits—thus justifying their great socioeconomic impact worldwide [6]. The main producers are Spain, Egypt, Turkey, Algeria, Italy, Greece, and Portugal; nevertheless, their production has undergone steady increases in such other regions as South America, Australia, and Middle East [7].

A great many table olive varieties exist in Portugal—namely, *Galega Vulgar*, *Cobrançosa*, *Mançanilha do Algarve*, *Carrasquenha*, *Cordovil de Castelo Branco*, *Cordovil de Serpa*, *Redondil*, and *Negrinha do Freixo* [3,8]; however, only the last one bears a protected denomination of origin (PDO) status. The probiotic potential of several of their endogenous strains has attracted interest by the scientific community—either lactic acid bacteria (LAB) or yeasts [9,10,11]. Although studies to date have focused on isolation and evaluation of probiotic features and benefits to human health of preselected strains of LAB, they are rather strain-specific—and adventitious strains are specifically related to the *terroir*; hence, screening of new probiotic candidates from understudied olive cultivars is in order [12]. Notwithstanding the fact that LAB strains, namely *Lactococcus* and *Lactiplantibacillus*, have been classified as “generally recognized as safe” (GRAS) by US Food and Drug Administration (FDA)—besides receiving a “Qualified Presumption of Safety” (QSP) status by European Food Safety Authority (EFSA)—novel strains isolated from native varieties are still to be subjected to food safety tests [2,13].

In addition to microbial safety, the gastrointestinal (GI) performance of candidate strains is also to be investigated, otherwise they may run the risk of losing viability as they cross the human gastrointestinal tract, and thus fail to exert a probiotic action once the host’s colon has been reached. By the time of administration, the probiotic should indeed show resistance to enzymes present in the oral cavity, such as amylases and lysozymes; the harsh environmental conditions prevailing in the stomach (low pH, presence of gastric juice with strong proteases) and in the intestine (exposure to pancreatin and bile salts); and also, be able to stand the mild heat shock caused by the internal body temperature [14].

This work was consequently aimed at characterizing strains isolated in advance from *Cobrançosa* olive cultivar (Trás-os-Montes, Portugal), with a focus on food safety for human consumption and survival in the GI tract; this effort was meant to select the best set of strains for a posteriori assessment in terms of probiotic performance in vivo, thus laying the groundwork for the eventual manufacture of a starter culture specific for table olive fermentation. Once a specific starter culture is put forward, the local manufacturers will be able to optimize growth conditions along table olive processing. The ultimate goal is to increase the market value of table olives by providing scientific evidence on their probiotic potential in complement to their intrinsic cultural value, and as reinforcement of their image as a food delicacy.

## 2. Materials and Methods

### 2.1. Sampling

Sampling of table olives from cultivar *Cobrançosa* was independently performed by selected producers in Trás-os-Montes*—*all of them following traditional protocols of spontaneous fermentation. Typically, this process follows two stages: (i) sweetening stage, where the olives are washed and added to spring water in different proportions, kept thereafter in water for 4*–*6 months, and subjected to periodic washing and addition of fresh water; and (ii) salting stage, where the water is no longer changed until the product is ready for selling to the market, additional salt is gradually added to the brine, up to 7*–*10% (*w*/*v*) by the time of selling. Sampling started on 19 November 2018 (right after harvest) and was concluded on 10 October 2019 (right after the product was ready for selling); an extra five samples were collected between these two dates. The harvest time does not coincide among all producers (and may differ by up to one month); this causes differences in drupe turning colour, and thus ripeness stage. The relation between isolates and fermentation time will be described later in the results section. Samples were collected as deep as possible from two fermentation drums per producer and immediately placed in previously labeled airtight, sterile containers; these were transported under refrigeration to our laboratory, and kept as such for no longer than 18 h prior to microbiological analysis.

### 2.2. Bacterial Strains and Growth Conditions

Nineteen LAB strains belonging to the genus *Lactiplantibacillus*, and species *paraplantarum* and *pentosus* were tested.; they were previously collected from *Cobrançosa* table olives and brines. In our laboratory, the strains were first screened for technological characteristics (i.e., ability to survive/grow under distinct salt concentrations, ability to survive/grow under high and low pH, capacity to degrade/assimilate oleuropein, and tendency to produce CO_2_). The strains, stored in 15% glycerol at −80 °C when not in use, were revived in de Man, Rogosa, and Sharpe (MRS) agar (VWR Chemicals, Leuven, Belgium) at 30 °C for 48 h; and then kept at 4 °C for up to one week, if meant to undergo experimentation. Just prior to testing, they were subcultured in MRS broth (VWR Chemicals), at 30 °C for 15 h without shaking (i.e., overnight incubation), so as to attain the stationary phase. A strain of *Lacticaseibacillus* (*Lc.*) *casei*, isolated in our lab from commercial probiotic yoghurt as well as a *Lactiplantibacillus* (*Lactobacillus*) *pentosus* strain B281 bearing probiotic properties, previously isolated from Greek table olives and kindly provided by Laboratory of Microbiology and Biotechnology of Foods, Department of Food Science and Human Nutrition, Agricultural University of Athens [15], were used as control strains from animal and vegetable origin, respectively.

### 2.3. Identification and Typing

For strain fingerprinting, LAB isolates were subjected to RAPD-PCR analysis using OPL5 primer (5′-ACGCAGGCAC-3′), as reported by Maldonado-Barragan et al. [16]. DNA extraction from pure cultures was performed using the GenElute™ Bacterial Genomic DNA kit (Sigma-Aldrich, St. Louis, MO, USA), according to manufacturer’s protocol. Amplifications were performed in an Uno Cycler (VWR) thermocycler. The NZYDNA Ladder VIII (NZYTech, Lisboa, Portugal) was run as a molecular size marker, and as reference lanes for band matching and inter-gel comparisons. Gels were visualized under UV light, and digitally captured using a gel documentation system (Cleaver Scientific, Rugby, UK). The RAPD profiles were analyzed visually, and further translated into binary matrices. Only reproducible bands representing amplicons between 200–5000 bp in size were considered. RAPD-PCR patterns were grouped by cluster analysis, using band-based Jaccard’s similarity coefficient and UPGMA algorithm. The similarity of band patterns was duly calculated, followed by clustering analysis. At least one representative strain of each profile was identified to the species level through amplification and sequencing of the 16S rRNA gene, using universal primers 27F (5′-GAGTTTGATCCTGGCTCAG-3′) and 1492R (5′-TACCTTGTTACGACTT-3′). The nucleotide sequences obtained were used to query the EzBioCloud database [17], and thus retrieved the closest strain—as per identification of isolates at the species level.

### 2.4. Assessment of Food Safety for Human Use

For each test, an aliquot (10 μL) of the overnight culture grown in MRS broth at 30 °C was spot-inoculated in duplicate on agar plates and incubated at 37 °C for 48 to 72 h. Overnight cultures of *Escherichia coli* ATCC 25,922 and *Staphylococcus aureus* ATCC 25923, grown in LB (Luria-Bertani) broth (VWR Life Science), were used for positive controls; whereas both *Lc. casei* (commercial probiotic yogurt) and *L. pentosus* (Greek probiotic table olives) strains were used for negative controls. In parallel to the safety tests, all strains were checked for viability using MRS agar plates for LAB strains, and plate count agar (PCA) for *E. coli* and *S. aureus* strains.

### 2.5. Mucin Degradation Test

To ascertain mucin degradation capacity, 1.5% (*w*/*v*) agar plates with 0.3% (*w*/*v*) mucin from porcine stomach M1778 (Sigma-Aldrich, St. Louis, Missouri, USA) and glucose 1% (*w*/*v*) (Sigma-Aldrich) were used [13]. Following incubation at 37 °C for 72 h, the mucin plates were flooded with 2 mL of 0.1% amido black 10B (Alfa Aesar, Haverhill, MA, USA), and prepared with 3.5 M glacial acetic acid (VWR Chemicals). After 30 min, mucin plates were washed twice with 1.2 M acetic acid, and halo formation around colonies (as indicator of pathogenicity) was recorded.

### 2.6. Hemolytic Activity Test

The hemolytic activity was assayed according to Benitez-Cabello et al. [18], with modifications, namely regarding utilization of blood agar base No. 2 supplemented with 5% defibrinated sheep blood (Biolife, Milan, Italy). The indicator of pathogenicity for hemolytic activity test was the development of a clear halo around the colonies on those plates.

### 2.7. DNase Activity Test

DNase activity was assessed after Anagnostopoulos et al. [19], via DNase Test Agar (Liofilchem, Province of Teramo, Italy), to check for production of DNase. Upon incubation at 37 °C for 24 h, pathogenicity was determined by observing medium transparency, once the DNA plates were flooded with HCl.

### 2.8. Assessment of Gastrointestinal Survival

The 19 strains were sequentially exposed to simulated GI fluids, as described in INFOGEST in vitro protocol [20,21], with modifications (see Table 1); simulated salivary fluid (SSF), simulated gastric fluid (SGF), and simulated intestinal fluid (SIF) were accordingly utilized. The experiments were performed in sterile 50 mL centrifuge tubes and incubated at 37 °C in a shaken water bath (100 rpm) to simulate peristaltic movements. Overnight LAB cultures grown in MRS broth at 30 °C (to achieve stationary phase), harvested by centrifugation (5110 rpm, 10–152 min, 5 °C), and washed twice in PBS (10 mM Na_2_HPO_4_, 1 mM KH_2_PO_4_, 137 mM NaCl, 2.7 mM KCl, pH 7.4), were resuspended in PBS to a final concentration of 10^9^ CFU/mL. The optical density (OD) at 600 nm was read to test for the final concentration of inoculum. Dilution and pour plating were performed for enumeration of colony-forming units (CFU) in the initial sample.

First, the oral phase was simulated by diluting (1:1) each bacterial cell suspension (4 mL) in SSF containing 0.1 mg/L of lysozyme. Subsequently, the pH was checked (and corrected, if necessary) to lie around 6.5 (by measuring the blank solution), and all solutions were duly incubated. After 2 min under shaking, the gastric phase was initiated by diluting the mixture (1:1) with pre-warmed SGF 1.25×, 13 mg of pepsin (2000 U in the final mixture), HCl 1 M, and sterile water—so as to attain a 1× concentration of SGF and pH 2.1. The mixture was incubated for 2 h at 37 °C, in an orbital shaker. The intestinal phase was started by diluting (1:1) the mixture with pre-warmed SIF (1.25×), NaOH 1 M, 20 mg pancreatin (100 U/mL in the final mixture), 3.2 mg of bile salts, and sterile water to attain a 1× concentration of SIF, pH 7.0. After 2 h of incubation, an aliquot (1 mL) of digested bacterial cells was decimally diluted down to 10^−6^ in sterile 0.85% (*v*/*v*) NaCl, plated on MRS agar in duplicate, and incubated at 37 °C for 48 h. Aliquots of overnight cultures were also tested to determine the number of CFU at time 0.

Although the optimum temperature of the wild strains isolated is ca. 30 °C, it should not be forgotten that olive trees in the open air are subjected to considerably higher temperatures during the period of maximum growth of their drupes; therefore, it is expected that our strains will be fully suitable for operation at 37 °C, i.e., the regular body temperature of humans.

The overall percent survival rate to gastrointestinal in vitro simulation was expressed by Equation (1):

(1) $$\mathrm{Survival}\;\mathrm{rate}\;\left(\%\right)=\frac{\log\;\left(\mathrm{CFU}\;\mathrm{after}\;\mathrm{digestion}\right)}{\log\;\left(\mathrm{CFU}\;\mathrm{before}\;\mathrm{digestion}\right)}\times 100$$

and CFU before digestion ($CFU_{initial})$ was determined following Equation (2):

(2) $$CFU_{initial}=CFU_{i}\times 10^{D}\times 10\times V_{suspension}$$

where *CFU_i_* denotes CFU before digestion, *D* required dilution, and *V_suspension_* initial volume before digestion.

Similarly, CFU after digestion (*CFU_final_*) was determined following Equation (3):

(3) $$CFU_{final}=CFU_{f}\times 10^{D}\times 10\times V_{total}$$

where *CFU_f_* denotes final counts, *D* required dilution, and *V_total_* volume by the end of the assay.

Bautista-Gallego et al. [22] reported that overall survival data are more realistic than results obtained upon application of a separate test for each stage of digestion; when applied, the sequential simulated gastrointestinal and pancreatic digestions allowed monitoring of the independent behavior of strains in response to each of them. Although prone to lead to a good independent choice, it might unfold an inappropriate global selection.

### 2.9. Statistical Analysis

One-way analysis of variance (ANOVA) was applied, followed by Tukey’s post hoc means multiple comparisons at 5% level of significance, with the aid of IBM SPSS 28.0 software (IBM, Armonk, NY, USA) and encompassed GI survival rates. Labeling of means with letters for comparison was ascribed as proposed by Piepho [23]. The same software was used to carry out principal component analysis (PCA), with oblique rotation (direct oblimin), applied to single out identification, table olive fermentation time, and survival rate, as well as for determination of Kaiser-Meyer-Olkin (KMO) measure, check of adequacy of sample size for analysis, and performance of Bartlett’s test of sphericity to unfold inter-correlations between variables. Additionally, Canoco™ V5.0 (Microcomputer Power, Ithaca, NY, USA) was used to produce both plot of loadings and plot of scores from PCA.

## 3. Results and Discussion

### 3.1. DNA-Fingerprinting and Identification

**Figure 1:**
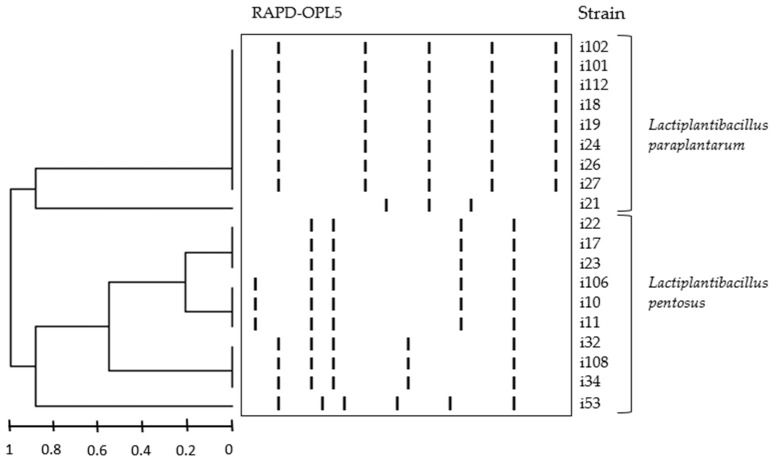
Dendogram generated from cluster analysis of digitalized OPL5 RAPD-PCR fingerprints of 19 LAB strains from *Cobrançosa* olive fermentations. Fingerprints were grouped by unweighted pair-group algorithm with arithmetic averages (UPGM).

Nineteen LAB strains were identified by combining RAPD-PCR and 16S rRNA sequencing; 16S rRNA sequences of these strains were deposited in the NCBI Gen Bank database, under the acession numbers OP376910 to OP376928. The dendogram generated with the pattern profile showed that the strains formed 6 different DNA fingerprints, belonging to species *L. pentosus* (4 fingerprints, 10 strains) and *L. paraplantarum* (2 fingerprints, 9 strains)*—*see Figure 1.

### 3.2. Assessment of Food Safety for Human Use

The positive control confirmed the expected behavior of pathogenic microorganisms*. E. coli* ATCC 25,922 revealed a brighter halo around its colony, thus unfolding its ability to degrade mucin. *S. aureus* ATCC 25,923 revealed hemolytic activity, as per a bright halo formed around each colony; and DNase activity (able to degrade DNA), for leaving the medium transparent.

All safety tests, encompassing *Cobrançosa* strains and negative strain control, turned negative; hence, none of the studied strains proved unsafe for human consumption. Similar results were obtained in several studies pertaining to table olives [7,24]. In order to exclude any false negative due to death throughout experimental development, all strains were grown in the corresponding growth medium.

### 3.3. Assesment of Gastrointestinal Survival

Resistance to gastrointestinal stress conditions is a major factor constraining use of microorganisms as probiotic agents. To be effective and confer health benefits, they must indeed be able to survive passage through the human mouth, stomach, and duodenum, and still be present to sufficient viable numbers to colonize the intestine [25]. Note that the data pertaining to the gastrointestinal transit were obtained in standard solutions, simple enough to minimize interference by lurking factors, yet able to mimic the physicochemical environment prevailing in the stomach and intestine. Although food microstructure obviously plays some role upon the transit patterns throughout the gastrointestinal tract, such a role breaks down as a more liquid form is attained, while mechanical action tends to dampen heterogeneities within the food matrix. In any case, the matrix effect (animal vs. vegetable) is believed to be minor compared to the chemical effects of pH, hydrolytic enzymes, and bile salts.

**Figure 2:**
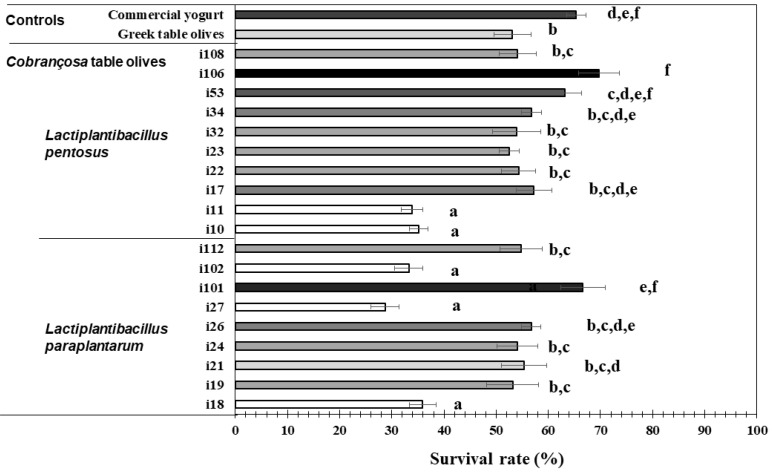
Gastrointestinal performance of native strains isolated from *Cobrançosa* table olives. Notes: Different letters represent significant differences (*p* < 0.05) between strains. *Lacticaseibacillus casei* strains were isolated from probiotic commercial yogurt. *Lactiplantibacillus pentosus* B281 strain was isolated from Greek probiotic table olives [15].

According to the results conveyed by Figure 2, the survival rates after facing the simulated adverse conditions prevailing in the GI tract varied from 28.72% ± 2.71 for strain i27 to 69.70% ± 3.88 for strain i106. Obtained from distinct RAPD profiles, six different homogenous subset groups (ANOVA, *p* = 0.000; Tukey, *p* < 0.005) could accordingly be pinpointed. Variability in survival rates among different species of *Lactiplanctibacillus* has been reported as well by Botta et al. [26].

The strains less resistant to GI conditions than those obtained from Greek probiotic table olives (51.13% ± 3.58) were i27, i102, i11, i10; hence, these strains were discarded, considering that their survival rate relative to control strains was low. On the other hand, strains exhibiting resistance to GI conditions similar to, or higher than probiotic strains from commercial yogurt (65.35% ± 1.85) were i53, i101 and i106, all from different RAPD profiles. Therefore, these strains should deserve special attention, namely complementary studies focused on adhesion to intestine epithelium, and in vivo protection against diseases and health conditions. The remaining 11 strains passing the above tests should be also subjected to further experimentation. Note that the protocol for GI survival used with the control strains was similar to that used with our own strains, so the effect of microstructure of the original food matrices (animal vs. vegetable) serving as vehicle thereof is likely marginal.

A few papers have reported on the capacity of the above species, isolated from table olives, to resist GI conditions. Despite the fact that not all such publications resort to analytical procedures directly comparable to the one selected for our study, it is generally considered that *L. pentosus* is a good probiotic candidate. Montoro et al. [27], Arroyo-Lopez et al. [28], Bautista-Gallego, Arroyo-López, Rantsiou, Jiménez-Díaz, Garrido-Fernández and Cocolin [22], and Guantario et al. [29] also developed work encompassing a number of strains of *L. Pentosus* and their results resemble our own data; such results on survival rate range between 40% and 80%, so they are consistent with those presented hereby. Considering *L. paraplantarum* isolated from Southern Portuguese table olives, Peres, Alves, Hernandez-Mendoza, Moreira, Silva, Bronze, Vilas-Boas, Peres and Malcata [9] reported survival rates up to 48%, a low figure compared to our results (between 29% and 67%); however, survival rates ranging from 40% to 60% for the strains of *L. paraplantarum* were reported elsewhere [30,31] pertaining to strains isolated from dairy products.

**Figure 3:**
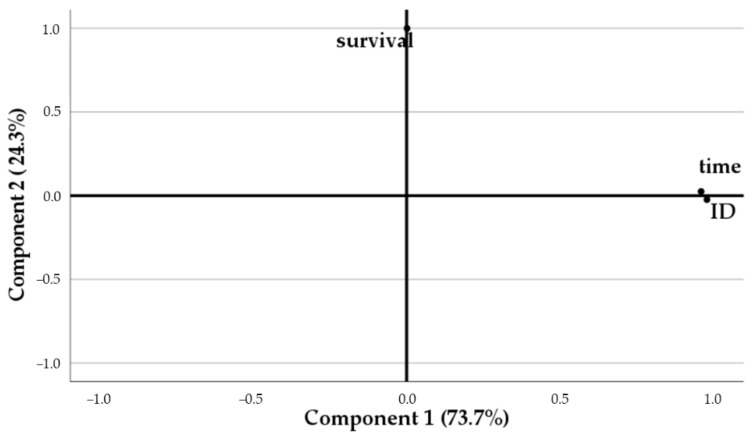
Plot of communalities from component extraction method, pertaining to survival rate throughout gastrointestinal tract.

Regarding PCA applied to survival rates, table olive fermentation time, and isolate identification, the KMO measure of 0.575 confirmed that the sampling size was adequate to extract significant information from factor analysis, with variables exhibiting significant correlation to each other, according to Bartlett’s test of sphericity (*p* = 0.000). Two components were accordingly selected from the scree plot (Figure 3) and were able to justify 98.0% of the total variance. Component 1 was highly correlated to table olive processing fermentation time (C1: 0.985, C2: 0.002) and strain identification (C1: 0.973, C2: 0.023), while component 2 was highly correlated to survival rate (C1: −0.001, C2: 1.000).

**Figure 4:**
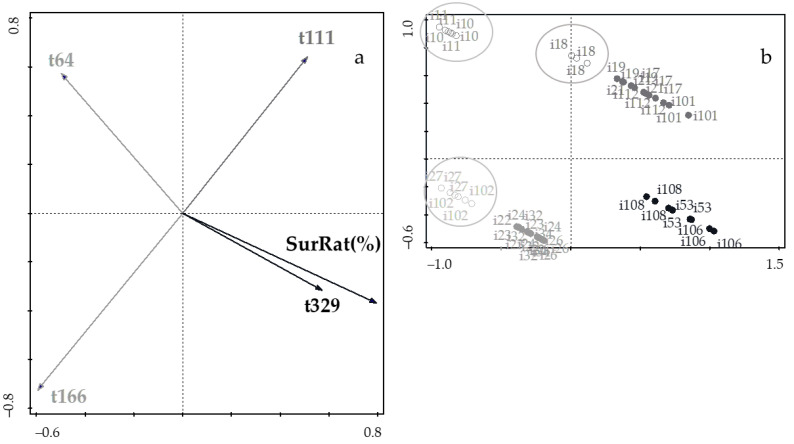
(**a**) Plot of loadings formed by the first two principal components from PCA, pertaining to probiotic characteristics associated with gastrointestinal survival and fermentation time; and (**b**) plot of scores encompassing strains isolated from *Cobrançosa* table olives.

Inspection of Figure 4a indicates that survival rate appears in the same position of the highest time of fermentation (sampling at 329 d), but opposite to the lowest time of fermentation (sampling at 64 d); these results are consistent with those in Figure 4b because LAB isolates bearing low values for time exhibited high values for survival rate, and vice versa. This is a consequence of ecological dominance by the most resistant strains. From the plots of scores (Figure 4b), four clusters were pinpointed as expected, in view of the underlying sampling/fermentation time. The circles indicate isolates previously discarded, based on explanations associated to Figure 2. No consistent rationale could be found for the lower resistance of strains i18, i27, and i102 considering that they belong to the cluster of 111 d and 166 d, respectively. Since this form of expressing results has not been utilized elsewhere, no comparison to parallel studies was possible at this time. In any case, it is remarkable that the best candidates for potential probiotics [6] are lactic acid bacteria isolated from *Cobrançosa* table olives by the end of the fermentation period for preceding ingestion by only a short delay.

### 3.4. Relevance and Pratical Implications

Besides responding to nutritional needs and providing sensory pleasure, table olives can provide protection against degenerative diseases and delay the incidence/severity of chronic health conditions as long as they are included in a balanced diet [6]. The growing demand for new probiotic foods has indeed stimulated the development of non-dairy products appropriate for addressing vegetarian trends and lactose-intolerance symptoms Concerted public health policies, triggered by budgetary restrictions as life expectancy becomes longer, will also urge a stronger focus on prevention rather than treatment [4]. Therefore, there is a window of opportunity in Mediterranean countries for table olives as carriers of probiotic strains, thus strengthening their added value [6]. World production for the 2019/20 campaign amounted to 2,961,000 tons, i.e., an increase of 5.5% compared to the previous campaign. Among the IOC member countries, Spain stands out for its weight in world production (15.5%), despite a 22% drop. Furthermore, Egyptian production has increased by 8% compared to the 2018/19 campaign, now contributing 22% of the world total [32]. Estimates for the 2020/21 campaign anticipate that production of table olives in Portugal will attain 21,000 ton/yr, with consumption increasing by 0.4% [33].

Commercial starters cannot stand the salty environment prevailing in olive brines, so useful strains must originate in the native microflora. The FAO/WHO has stressed that probiotic traits are typically strain-specific, so candidates are to be investigated ab initio, especially when genera beyond *Lactiplantibacillus* are at stake [34]. Therefore, an effective strategy requires sequential screening (of existing strains) for safety for human ingestion and resistance to simulated GI tract before they are ready for validation based on probiotic performance. Following in loco testing, a putative industrial process based on the best strains should be assessed from social, environmental, and economic points of view, thus supporting an acceptable in orbi process. This will ultimately guarantee a clean industrial technology via preliminary evaluation of environmental and social impacts, while assuring an economically feasible approach.

## 4. Conclusions

Nineteen adventitious strains isolated from *Cobrançosa* table olives were identified by combining RAPD-PCR and 16S rRNA sequencing. The dendogram generated with the pattern profile showed that the strains formed 6 different DNA fingerprints, belonging to species *L. pentosus* (four fingerprints, 10 strains) and *L. paraplantarum* (two fingerprints, nine strains).

Three tests were performed to evaluate the food safety potential of these strains. All strains were actually safe for human consumption, as per the negative results obtained for mucin degradation, hemolytic activity, and DNase activity.

*L. paraplantarum* i27 exhibited the lowest survival rate (29%). Thirteen strains exhibited higher survival rates to gastrointestinal conditions than the Greek probiotic table olive strain (53%); and from these, 11 strains exhibited lower survival rates than a commercial probiotic yogurt strain (65%). The other two strains showed the best resistance to the gastrointestinal system (i.e., 67% for *L. paraplantarum* i101, and 70% for *L. pentosus* i106).

Viability at the end of gastrointestinal digestion is the most critical parameter for probiotic activity, as it determines the eventual impact of probiotic bacteria upon consumers’ health. The gastrointestinal performance of our strains improved, in general, as fermentation time increased. Based on the above findings, the 14 strains, considered safe and exhibiting good gastrointestinal performance, appear suitable for more refined, complementary steps meant for the confirmation of their probiotic potential.

## Figures and Tables

**Table 1:** Simulated sequential steps of gastrointestinal digestion assay based on modified INFOGEST protocol (semi-dynamic test).

Oral		Gastric		Intestinal	
Duration (min)					
2 *		120		120	
Fixed Volumes (mL)					
Salivary fluid (SSF) (5/4)	3.2	Liquid food	8.0	Liquid food	16.0
CaCl_2_ 0.3 M	0.020	Gastric juice (SGF) (5/4)	6.4	Duodenal juice (SIF) (5/4)	6.8
Salivary amylase solution	0.4	Pepsin solution	0.4	Pancreatin	4.0
		CaCl_2_ 0.3 M	0.004	Bile	2.0
				CaCl_2_ 0.3 M	0.032
Variable volumes (mL)					
		HCl 1 M to pH 3.0	0.740	Acid/base 1 M to pH 7.0	0.350
Water	0.380	Water	0.456	Water	2.818
Total	8.0	Total	16.0	Total	32.0

* Time amylase is active in food; quantity of inoculum (9–10 log): 4.0 mL.

## Data Availability

Data is contained within the article.

## References

[1] Bigliardi B., Filippelli S. (2022). A review of the literature on innovation in the agrofood industry: Sustainability, smartness and health. Eur. J. Innov. Manag..

[2] Koirala S., Anal A.K. (2021). Probiotics-based foods and beverages as future foods and their overall safety and regulatory claims. Future Foods.

[3] Albuquerque T.G., Costa H.S., Oliveira M.B.P.P. (2019). An overview of Portuguese olive oils and table olives with Protected Designation of Origin. Eur. J. Lipid Sci. Technol..

[4] De Bellis P., Sisto A., Lavermicocca P. (2021). Probiotic bacteria and plant-based matrices: An association with improved health-promoting features. J. Funct. Foods.

[5] Bulut Albayrak Ç., Duran M. (2022). Worldwide research tendencies on probiotics in food science: 1993 to 2021. Br. Food J..

[6] Anagnostopoulos D.A., Tsaltas D. (2022). Current status, recent advances, and main challenges on table olive fermentation: The present meets the future. Front. Microbiol..

[7] Perpetuini G., Prete R., Garcia-Gonzalez N., Khairul Alam M., Corsetti A. (2020). Table olives more than a fermented food. Foods.

[8] Pires-Cabral P., Nunes P., Barros T., Quintas C. (2018). Physicochemical, nutritional and microbiological characteristics of traditional table olives from Southern Portugal. Emir. J. Food Agric..

[9] Peres C.M., Alves M., Hernandez-Mendoza A., Moreira L., Silva S., Bronze M.R., Vilas-Boas L., Peres C., Malcata F.X. (2014). Novel isolates of lactobacilli from fermented Portuguese olive as potential probiotics. LWT Food Sci. Technol..

[10] Oliveira T., Ramalhosa E., Nunes L., Pereira J.A., Colla E., Pereira E.L. (2017). Probiotic potential of indigenous yeasts isolated during the fermentation of table olives from Northeast of Portugal. Innov. Food Sci. Emerg. Technol..

[11] Pereira E.L., Ramalhosa E., Borges A., Pereira J.A., Baptista P. (2015). YEAST dynamics during the natural fermentation process of table olives (*Negrinha de Freixo cv.*). Food Microbiol..

[12] Wang J., Wang J., Yang K., Liu M., Zhang J., Wei X., Fan M. (2018). Screening for potential probiotic from spontaneously fermented non-dairy foods based on in vitro probiotic and safety properties. Ann. Microbiol..

[13] Abouloifa H., Rokni Y., Bellaouchi R., Ghabbour N., Karboune S., Brasca M., Ben Salah R., Chihib N., Saalaoui E., Asehraou A. (2020). Characterization of Probiotic Properties of Antifungal *Lactobacillus* Strains Isolated from Traditional Fermenting Green Olives. Probiotics Antimicrob. Proteins.

[14] de Melo Pereira G.V., de Oliveira Coelho B., Magalhaes Junior A.I., Thomaz-Soccol V., Soccol C.R. (2018). How to select a probiotic? A review and update of methods and criteria. Biotechnol. Adv..

[15] Bonatsou S., Tassou C.C., Panagou E.Z., Nychas G.E. (2017). Table olive fermentation using starter cultures with multifunctional potential. Microorganisms.

[16] Maldonado-Barragan A., Caballero-Guerrero B., Lucena-Padros H., Ruiz-Barba J.L. (2013). Induction of bacteriocin production by coculture is widespread among plantaricin-producing *Lactobacillus plantarum* strains with different regulatory operons. Food Microbiol..

[17] Yoon S.H., Ha S.M., Kwon S., Lim J., Kim Y., Seo H., Chun J. (2017). Introducing EzBioCloud: A taxonomically united database of 16S rRNA gene sequences and whole-genome assemblies. Int. J. Syst. Evol. Microbiol..

[18] Benitez-Cabello A., Calero-Delgado B., Rodriguez-Gomez F., Garrido-Fernandez A., Jimenez-Diaz R., Arroyo-Lopez F.N. (2019). Biodiversity and multifunctional features of lactic acid bacteria isolated from table olive biofilms. Front. Microbiol..

[19] Anagnostopoulos D., Bozoudi D., Tsaltas D. (2018). Enterococci isolated from Cypriot green table olives as a new source of technological and probiotic properties. Fermentation.

[20] Minekus M., Alminger M., Alvito P., Ballance S., Bohn T., Bourlieu C., Carriere F., Boutrou R., Corredig M., Dupont D. (2014). A standardised static *in vitro* digestion method suitable for food—An international consensus. Food Funct..

[21] Mulet-Cabero A.I., Egger L., Portmann R., Menard O., Marze S., Minekus M., Le Feunteun S., Sarkar A., Grundy M.M., Carriere F. (2020). A standardised semi-dynamic *in vitro* digestion method suitable for food—An international consensus. Food Funct..

[22] Bautista-Gallego J., Arroyo-López F.N., Rantsiou K., Jiménez-Díaz R., Garrido-Fernández A., Cocolin L. (2013). Screening of lactic acid bacteria isolated from fermented table olives with probiotic potential. Food Res. Int..

[23] Piepho H.P. (2018). Letters in mean comparisons: What they do and don’t mean. J. Agron..

[24] Portilha-Cunha M.F., Macedo A.C., Malcata F.X. (2020). A review on adventitious lactic acid bacteria from table olives. Foods.

[25] Soares M.B., Martinez R.C.R., Pereira E.P.R., Balthazar C.F., Cruz A.G., Ranadheera C.S., Sant’Ana A.S. (2019). The resistance of *Bacillus*, *Bifidobacterium*, and *Lactobacillus* strains with claimed probiotic properties in different food matrices exposed to simulated gastrointestinal tract conditions. Food Res. Int..

[26] Botta C., Langerholc T., Cencic A., Cocolin L. (2014). *In vitro* selection and characterization of new probiotic candidates from table olive microbiota. PLoS ONE.

[27] Montoro B.P., Benomar N., Lavilla Lerma L., Castillo Gutierrez S., Galvez A., Abriouel H. (2016). Fermented Alorena table olives as a source of potential probiotic *Lactobacillus pentosus* strains. Front. Microbiol..

[28] Arroyo-Lopez F.N., Blanquet-Diot S., Denis S., Thevenot J., Chalancon S., Alric M., Rodriguez-Gomez F., Romero-Gil V., Jimenez-Diaz R., Garrido-Fernandez A. (2014). Survival of pathogenic and *lactobacilli* species of fermented olives during simulated human digestion. Front. Microbiol..

[29] Guantario B., Zinno P., Schifano E., Roselli M., Perozzi G., Palleschi C., Uccelletti D., Devirgiliis C. (2018). *In vitro* and *in vivo* selection of potentially probiotic Lactobacilli from Nocellara del Belice table olives. Front. Microbiol..

[30] Tulini F.L., Winkelstroter L.K., De Martinis E.C. (2013). Identification and evaluation of the probiotic potential of *Lactobacillus paraplantarum* FT259, a bacteriocinogenic strain isolated from Brazilian semi-hard artisanal cheese. Annaerobe.

[31] Kalhoro M.S., Visessanguan W., Nguyen L.T., Anal A.K. (2019). Probiotic potential of *Lactobacillus paraplantarum* BT-11 isolated from raw buffalo (*Bubalus bubalis*) milk and characterization of bacteriocin-like inhibitory substance produced. J. Food Process. Preserv..

[32] IOOC IOC News: Table Olives—Provisional 2019/20 Campaign and Estimate for 2020/21. https://www.internationaloliveoil.org/.

[33] IOOC IOC Figures: Production of Table Olives. https://www.internationaloliveoil.org/.

[34] Berkes E., Liao Y.H., Neef D., Grandalski M., Monsul N. (2020). Potentiated *in vitro* probiotic activities of *Lactobacillus fermentum* LfQi6 biofilm biomass *versus* planktonic culture. Probiotics Antimicrob. Proteins.

